# Low β2 Main Peak Frequency in the Electroencephalogram Signs Vulnerability to Depression

**DOI:** 10.3389/fnins.2016.00495

**Published:** 2016-11-02

**Authors:** Damien Claverie, Chrystel Becker, Antoine Ghestem, Mathieu Coutan, Françoise Camus, Christophe Bernard, Jean-Jacques Benoliel, Frédéric Canini

**Affiliations:** ^1^Département Neurosciences and Contraintes Opérationnelles, Institut de Recherche Biomédicale des ArméesBrétigny-sur-Orge, France; ^2^Sorbonne Universités, Pierre and Marie Curie University Univ Paris 06, INSERM, CNRS, Neurosciences Paris Seine - Institut de Biologie Paris Seine (NPS - IBPS), Site Pitié-SalpêtrièreParis, France; ^3^Institut National de la Santé et de la Recherche Médicale, U1130Paris, France; ^4^Centre National de la Recherche Scientifique, UMR8246Paris, France; ^5^Université Paris Descartes, Sorbonne Paris Cité, Faculté de MédecineParis, France; ^6^Aix Marseille Univ., INSERM, INS, Inst. Neurosci. Syst.Marseille, France; ^7^AP-HP, Hôpital de la Pitié-Salpêtrière, Service de Biochimie Endocrinienne et OncologiqueParis, France; ^8^Ecole du Val de GrâceParis, France

**Keywords:** BDNF, EEG, vulnerability to depression, Beta 2, peak frequency, social defeat, stress, biomarker

## Abstract

**Objective:** After an intense and repeated stress some rats become vulnerable to depression. This state is characterized by persistent low serum BDNF concentration. Our objective was to determine whether electrophysiological markers can sign vulnerability to depression.

**Methods:** Forty-three Sprague Dawley rats were recorded with supradural electrodes above hippocampus and connected to wireless EEG transmitters. Twenty-nine animals experienced four daily social defeats (SD) followed by 1 month recovery. After SD, 14 rats had persistent low serum BDNF level and were considered as vulnerable (V) while the 15 others were considered as non-vulnerable (NV). EEG signals were analyzed during active waking before SD (Baseline), just after SD (Post-Stress) and 1 month after SD (Recovery).

**Results:** We found that V animals are characterized by higher high θ and α spectral relative powers and lower β2 main peak frequency before SD. These differences are maintained at Post-Stress and Recovery for α spectral relative powers and β2 main peak frequency. Using ROC analysis, we show that low β2 main peak frequency assessed during Baseline is a good predictor of the future state of vulnerability to depression.

**Conclusion:** Given the straightforwardness of EEG recordings, these results open the way to prospective studies in humans aiming to identify population at-risk for depression.

## Introduction

Depression is thought to be the result of interaction effects between the individual and the environment (Harro, 2013). The diathesis-stress model provides a conceptual framework to understand the occurrence of depression. The diathesis represents the vulnerability of a given individual (Bernard, 2016). Such vulnerability includes numerous factors, such as genetic (Sullivan et al., 2000) and environmental ones (Lueboonthavatchai, 2009). Stress is the triggering event leading to the pathological state. Prevention of depression requires an early detection of vulnerable subjects, i.e., individuals presenting a high risk to develop depression when exposed to a stressful event. Finding biomarkers of vulnerability to depression is a key issue to identify high risk subjects, who do not present clinical signs, in order to open the way to preventive treatment.

Social defeat is one of the most intense life events inducing high stress levels in humans (Björkqvist, 2001) and animals (Koolhaas et al., 1997). Social defeat can change diathesis without necessarily triggering depression, a concept that we have validated in an animal model (Blugeot et al., 2011; Becker et al., 2015; Bouvier et al., 2016). Five days following four daily social defeats (SD), all rats are characterized by low serum Brain Derived Neurotrophic Factor (sBDNF) levels. One month later, sBDNF levels remain low in 40–50% of the animals, whilst they recover in the remaining population. At this time point, there is no difference in behavior and blood corticosterone concentration in rats with normal and low sBDNF levels. When exposed to a second challenge, such as chronic mild stress (Blugeot et al., 2011; Bouvier et al., 2016) or kainic acid injection (Becker et al., 2015), only animals with low sBDNF levels (hereafter called vulnerable—V) develop a depression-like profile. The latter was characterized by an increased immobility time in a forced swimming test, decreased sucrose consumption, and blunted weight gain, an increase in blood cortisol levels and adrenal weight, characteristic features of hypothalamic-pituitary-adrenal axis hyperactivity. Neuroanatomical alterations are also present, including reduced hippocampal volume, reduced hippocampal neurogenesis, dendritic retraction, and decrease in spine density in CA3 pyramidal cells, and amygdala hypertrophy (Blugeot et al., 2011; Becker et al., 2015; Bouvier et al., 2016). None of these features are found in animals in which sBDNF recovered to control levels (Blugeot et al., 2011). The latter are called non-vulnerable (NV) or resilient. In such experimental conditions, sBDNF concentration can be used as a predictive biomarker of vulnerability to depression after SD with a sensibility of 81% and a specificity of 89% (Blugeot et al., 2011).

A major stressful event is necessary to identify the vulnerable population based upon sBDNF levels. Ideally, in the context of preventive treatments, it would be important to identify the future vulnerable population *before* SD. Before SD, all future V and NV animals present identical sBDNF levels. Yet, they are not biologically identical, since they will react differently to SD. The goal of the present study was to identify an early biomarker, predictive of the vulnerability to depression, which can be used before social stress. We focused on the properties of electroencephalographic (EEG) signals as depressive patients show EEG abnormalities when they are awake (for review Pollock and Schneider, 1990; Olbrich and Arns, 2013); these abnormalities being considered as biomarkers (Steiger and Kimura, 2010). Awake depressive patients have an increase (Knott et al., 2001) or decrease (Lubar et al., 2003) in mean frequency of the spectral power calculated on the whole EEG spectrum. The analysis of the spectral power of specific bands during resting state reported conflicting data on the δ [increase (Kwon et al., 1996; Begić et al., 2011) or not (Pollock and Schneider, 1990; Volf and Passynkova, 2002)], θ [increase (Kwon et al., 1996; Ricardo-Garcell et al., 2009; Grin-Yatsenko et al., 2010; Jaworska et al., 2012) or not (Pollock and Schneider, 1990; Volf and Passynkova, 2002)], and α [increase (Pollock and Schneider, 1990; Ricardo-Garcell et al., 2009; Grin-Yatsenko et al., 2010; Jaworska et al., 2012) or not (Volf and Passynkova, 2002; Begić et al., 2011)] bands; whilst the β band consistently showed an increase (Pollock and Schneider, 1990; Knott et al., 2001; Grin-Yatsenko et al., 2010; Begić et al., 2011). The main abnormality reported in awake depressive patients is an inter-hemispheric asymmetry with a global left frontal hypoactivity (Fingelkurts et al., 2006) and right posterior hypoactivity (Bruder et al., 1997; Manna et al., 2010). The most described asymmetry in left frontal and right posterior areas is an increased power in the α band (Reid et al., 1998; Kentgen et al., 2000). In addition, β and θ asymmetries have also been described (Roemer et al., 1992). Interestingly, the asymmetry in the α band has been reported not only in depression but also in subjects free from symptoms but having at least one parent suffering from depression (Bruder et al., 2005). Further, the hemispheric asymmetry in the α band is independent of the affective state as it is observed in non-depressed subjects (Bruder et al., 2005) as well as remitted depressive patients (Henriques and Davidson, 1990). Since EEG abnormalities can be observed in depressed patients, in remitted patients and in subjects considered as prone to depression, we thus investigated whether V and NV animals could be distinguished by differences in EEG properties assessed in the different frequency bands after SD or even retrospectively, before SD and before sBDNF signs of vulnerability. In order to be congruent with data obtained in humans, we considered in our study only the state of vigilance of active waking.

## Materials and methods

### Animals

The investigation was performed in 43 male Sprague–Dawley rats (Janvier Laboratories, France) weighing 125–130 g upon arrival at the laboratory. They were housed in animal facilities equipped with regularly spaced, sound-proof, temperature-controlled compartments supplied with filtered air (Enceinte Autonome d'Animalerie; A110SP, Thermo Electron). The environment was controlled: light/dark cycle control (12 h–12 h dark-light cycle with lights on at 07:00 a.m.), ambient temperature (21 ± 1°C) and relative humidity (50 ± 10%). The rats had *ad libitum* access to food and water. Male wild-type Groningen (WTG) rats weighing 550–600 g (Groningen, Netherlands) were used as resident rats in confrontation encounters (de Boer et al., 2003). Procedures involving animals and their care were performed in accordance with institutional guidelines conforming to national and international laws and policies (council directive #87–848, October 19, 1987, Ministère de l'Agriculture et de la Forêt, Service Vétérinaire de la Santé et de la Protection Animale, permissions #75–1178 to J.J.B., 07-02-2013 to D.C.) and were approved specifically by our ethics committee: Inserm-European Community Agreement of sept 22nd 2010 to C.B.

### Experimental design

**Figure 1:**
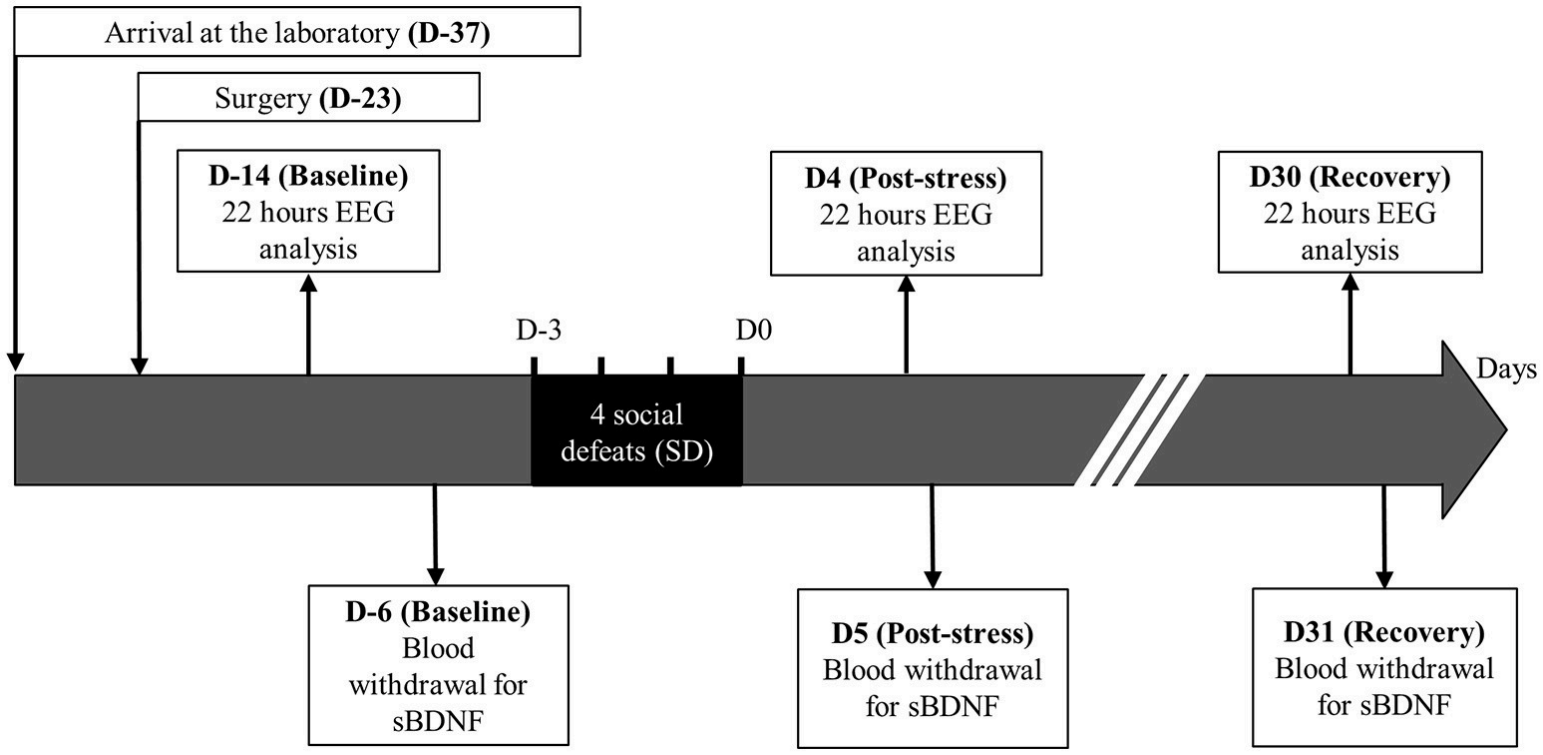
**Experimental protocol**.

The 43 rats followed the same experimental time course (Figure 1). At laboratory arrival, the animals belonging to the same litter were housed in the same home cage for 4 days. They were then transferred to individual cages (length, 45 cm; width, 25 cm; height, 17 cm) for 10 days. Thereafter, they were surgically equipped with a telemetric recorder [TL11M2-F20-EET, DataScience International (DSI), Mineapolis, USA]. They were allowed a 20-days period for surgery recovery. At the end of that period, the animals were randomly distributed into one of the two following groups: the stressed rats (Stress, *n* = 29) were exposed to 4 daily successive social defeat (SD) while Sham rats (Sham, *n* = 14) were not exposed to social defeat. After SD, the animals stayed 1 month in their home cage for stress recovery. Stressed and Sham rats were handled separately throughout the investigation during 1 h each day, except the 4 days of SD for the stressed animals which were exposed to 1 h social defeat. Common remaining non-handled hours (22 h) were used to perform telemetric recordings.

During this time course, 3 experimental times were isolated: the control time (Baseline), the post-stress time (Post-stress), and the recovery time (Recovery). For Baseline, the EEG recording was carried out 9 days after surgery to ensure a complete recovery and an EEG signal stabilization (Gage et al., 2012). The blood sampling for sBDNF assay was done 8 days after, that is 3 days before SD in order to be congruent with our previous results (Blugeot et al., 2011; Becker et al., 2015; Bouvier et al., 2016). For Post-stress, the EEG recording and the blood withdrawal were carried out 4 and 5 days after the last SD, respectively. For Recovery, the EEG recording and the blood withdrawal were practiced 30 and 31 days after the end of SD, respectively. The difference in timing between EEG recording and blood withdrawal was justified by avoiding any interaction effect between assessments.

### Social defeat procedure

The social defeat procedure was performed as previously reported (Becker et al., 2001; Andre et al., 2005; Blugeot et al., 2011). The same pairs (resident, intruder) were used for the four daily conditioning sessions. Each social defeat lasted 45 min and was divided into two consecutive periods. During period I (30 min), intruders were placed individually in a protective cage inside the resident animal's home cage. The protective cage allowed unrestricted visual, auditory, and olfactory contact with the resident but prevented any close physical contact. During period II (15 min), the protective cage was removed, allowing physical confrontation between the resident and the intruder. There were 3–4 confrontations lasting roughly 10 s in each social defeat exposure. During each confrontation, the intruding animal was always dominated (defeated) by the resident rat. Animals wounded during the social defeat procedure (2%) were excluded from the experiment. Telemetric measurements and blood collections were only performed for intruders.

### Surgery

The rats were implanted with the TL11M2-F20-EET telemetric transmitter allowing core body temperature (T_c_), locomotion and EEG assessments (DataScience, Mineapolis, USA). The EEG was assessed as a right parietal electrocorticogram in front of right hippocampus, a brain area involved in depression with functional and structural alterations (Khalid et al., 2014). Moreover, we previously demonstrated that morphology of CA3 neurons of hippocampus was chronically altered in V animals comparatively to NV ones (Blugeot et al., 2011; Bouvier et al., 2016). Parietal electrocorticogram and hippocampus LFP are highly interconnected and synchronized (Qin et al., 1997). The transmitter was inserted in the rats' abdominal cavity under deep anesthesia (Ketamine, Vétoquinol, 100 mg/kg *i.p.*; Xylazine 2%, Bayer, 10 mg/kg *i.p.*). A sagittal abdominal incision was made and the telemetric device was placed in the peritoneal cavity. Muscles and the skin were then stitched up. The first electrode pair assembled a reference electrode secured above cerebellum and a recording electrode secured on parietal cortex just above right hippocampus CA1 (−4.0 mm posterior, +2.0 mm lateral; 43 rats). The second electrode pair was implanted either into cervical muscles to obtain electromyogram (EMG, 6 rats) or on the skull symmetrically to the previous pair (−4.0 mm posterior, −2.0 mm lateral; 37 rats). The brain electrodes were maintained in the skull using stainless screws and dental cement (UNIFAST Trad, GC America). Electrodes used for EMG were stitched up in the muscle. The rats received antibiotic (Ampicilline 5G, Cadril, 0.5 g/kg, *s.c.*) during 3 consecutive days following the surgery and were allowed 20 days to recover from surgery.

### Telemetric assessments

The RPC-1 receiver plates and Data Exchange matrix were connected to a computer running on ART-gold software 3.1 (Data Sciences International, Saint-Paul, MN, USA). We recorded right hippocampus EEG, T_c_, and locomotion in all animals and additionally either EMG (6 rats) or left hippocampus EEG (37 rats). The 22 h of recording were split into 5 s epochs (5 s-epochs) to analyze the concomitant telemetric variables (i.e., T_c_, locomotion, EEG).

The EEG and EMG signals were recorded at a sampling rate of 500 Hz. Only the right EEG signal above hippocampus CA1 was analyzed. Analyzes were carried out only on artifact free 5 s-epochs. According to three reference methods (Tong and Thakor, 2009), epochs were considered as artifacted when at least one value of the signal was missing, or when the absolute variation of the signal slope was greater than 0.075 μV/ms, or when the absolute value of the signal was >5 standard deviations of the mean. After artifacted epochs rejection, the EEG power spectral analysis was performed in each non-artifacted epoch using a Fast Fourier Transform algorithm with Welch estimation (500 points, hamming window, and 50% overlap) perform with Matlab (Mathworks, r2013b version). The telemetric bandwidth being 1–50 Hz according to the manufacturer, only the following bands were considered (δ: 1.5–4 Hz, Low θ: 4–6.5 Hz, High θ: 6.5–9.5 Hz, α: 9.5–12 Hz, β1: 13–18 Hz, β2: 18–25 Hz, Slow γ: 25–48 Hz). The absolute power of each band was calculated from EEG spectrum as the area under the spectrum curve. The relative power of each band was expressed in ratio of the total power spectrum in each epoch. When present, the frequency of maximal peak power was identified in each band by an integrated function of Matlab (findpeaks function). This frequency was called main peak frequency. Numbers of active wake epochs where at least one peak was detected were reported in Supplementary Table 1. The variables calculated from EEG (i.e., mean relative powers and mean main peak frequencies in each band) were calculated in each animal as the averaged value of all the 5 s-epochs corresponding to active waking.

Since T_c_ changes after SD (Meerlo et al., 1996) and during depression (Shiromani et al., 1991), T_c_ was assessed throughout the experiment. The T_c_ signal was recorded at a sampling rate of 250 Hz. A mean value was calculated for each epoch. The T_c_ was then expressed in each animal and in each time (Baseline, Post-stress, and Recovery) as the hourly mean values of the 5 s-epochs of active waking in order to take into account the circadian variation of T_c_ (see Supplementary Figure 1).

Since locomotion is enhanced in animals expressing a depression-like profile (Strekalova and Steinbusch, 2010) and may affect EEG patterns (Wells et al., 2013), we assessed their locomotion within their home cage. The locomotion signal was recorded at a sampling rate of 1 Hz and was assessed as a variation of signal strength of the telemetric recording detected by the DSI antennas. This method is validated (DSI device specifications; Michel et al., 2010), and assumed to be related to the animal speed, 2 AU/s corresponding to 1 cm/s (Studholme et al., 2013; Vivanco et al., 2013). The spontaneous locomotion activity (SLA) was calculated as the sum of the activities recorded during each epoch expressed in AU. Therefore, SLA = 2 corresponds to one burst of locomotion during 1 s at a speed of 1 cm/s. The SLA was then expressed in each animal as the averaged value of all the 5 s-epoch corresponding to active waking (see Supplementary Figure 2).

### Definition of active waking

We determine the SLA threshold of active waking *vs.* inactive waking and sleep in a preliminary analysis carried out on the Baseline 22-h recording of the 6 animals equipped with EMG electrodes. We determine the vigilance level and the SLA value in a high number of 5 s-epochs in order to validate the use of SLA as a biomarker of active wake. The vigilance analysis, i.e., repartition of epochs into wake, slow wave sleep and paradoxical sleep stages, was performed visually in 5 s-epochs in a blind-to-treatment manner using a presentation software running on Matlab. Sleep and waking represented 42.9 ± 1.6 and 57.0 ± 1.4% of the time, respectively. The waking was further divided into active (15.1 ± 0.5%) and inactive (41.9 ± 0.9%) waking. A SLA ≥ 1 AU was rarely observed during sleep (298 epochs out of 15,433 epochs), but were predominant during wake (15,135 out of 15,433 epochs). Therefore, a SLA ≥ 1 AU threshold provides good specificity (99.3%) and predictive power (98.1%) for active waking. Increasing the threshold value did not improve the specificity and the positive predictive power of SLA for active waking.

To consider the state of vigilance of active waking, we only kept for analyzes the non-artifacted 5 s-epochs with a SLA ≥ 1 AU found in the 22-h recordings.

### Control of interaction between locomotion and EEG

The influence of motor activity on EEG waves has been controlled. We did not observe any significant difference of SLA values among groups (see Supplementary Figure 2). In the literature, the influence of locomotion on EEG was reported for the high θ wave only for speed motor activities exceeding 10 cm/s (Wells et al., 2013). In our study, motor activity did not reach higher speed than 8 cm/s. This can be explained by experimental conditions: an ecological recording in home cage and a probably not goal-oriented. Actually, there was no difference in theta waves' power or peak frequency according to locomotion speed both on V and NV animals (see Supplementary Figure 3).

### Active wake main peak repartition into β2 band

The repartition of the β2 main peaks during all 5 s-epochs of active waking during Baseline recording was expressed in each future vulnerability group (V and NV) in function of frequency bins. The β2 band was separated into 7 bins (1 bin by hertz) to represent the β2 main peak frequency repartition by a histogram. The mean value and standard deviation value of the number repartition of β2 main peak frequency into each bin were determined for stressed animals. In order to better compare the repartition, each bin frequency was expressed in z-score for each animal [(value-all group mean of the bin)/all group standard deviation of the bin]. For each future vulnerability group (V and NV) mean values ± standard error of the mean of z-scores were calculated.

### Blood withdrawal and BDNF assay

Blood samples (200 μl) were taken from the tail vein between 11 a.m. and 12 p.m. from conscious rats at Baseline, Post-Stress, and Recovery (Blugeot et al., 2011).

The samples were centrifuged to separate off the serum, which was stored at −20°C until BDNF analysis. BDNF concentrations were determined at a dilution of 1:25, with a commercial BDNF assay (Promega Corporation, Madison, Wisconsin, USA), in 96-well plates (Corning Costar® EIA plate, New York, USA), according to the manufacturer's instructions. Assay limit of detection of this commercial BDNF assay kit is of 7.8 pg/mL and the limit of quantification is of 15.6 pg/mL. The intra-assay precision of the BDNF for our concentrations given by the manufacturer is between 2.2 and 2.9% of coefficient of variance. Our samples were tested in duplicates. The BDNF concentration was expressed in ng/mL. The determination of sBDNF levels was performed in one run when all samples have been collected.

### Statistical analysis

Statistical analyses were performed with Statistica software v7.1 (StaSoft-France, Maisons-Alfort, France).

Two *K*-mean Group effects performed on stressed animals were based on sBDNF values at Baseline and Recovery to determine the V vs. NV groups. The k-mean algorithm distributed the animals into a given number of subgroups in a way that minimize the intragroup variability and maximize the intergroup variability. In our case, we chose 2 subgroups (i.e., V and NV animals).

Time course was analyzed using repeated measures Analysis of Variance (ANOVA; Time effect) with conditioning effects (Group and Group^*^Time effects). Time effects corresponded to the effects of measure repetition (i.e., Baseline, Post-Stress, and Recovery). Group effects corresponded to the effects of group classification (i.e., V and NV subgroups). Group^*^Time effects corresponded to the combined effects of the time and the group classification. When ANOVA revealed a significant effect, a *post-hoc* Bonferroni tests for all couples were carried out. Correlations within subgroups were done using multiple correlation tests. The significance level was set at *p* < 0.05. Data are presented as means ± standard error of the mean (SEM).

To determine whether observed differences could represent biomarker, data underwent Receiver Operating Characteristic (ROC) curve analyses. The Area Under the ROC Curve (AUC) provides a measure of overall performance of the classifier. AUC with corresponding 95% Confidence Intervals (CI_95_) were calculated with the statistical software package SPSS 22 for Windows (SPSS Inc., Chicago, USA).

## Results

### Identification of vulnerable and non-vulnerable animals

**Figure 2:**
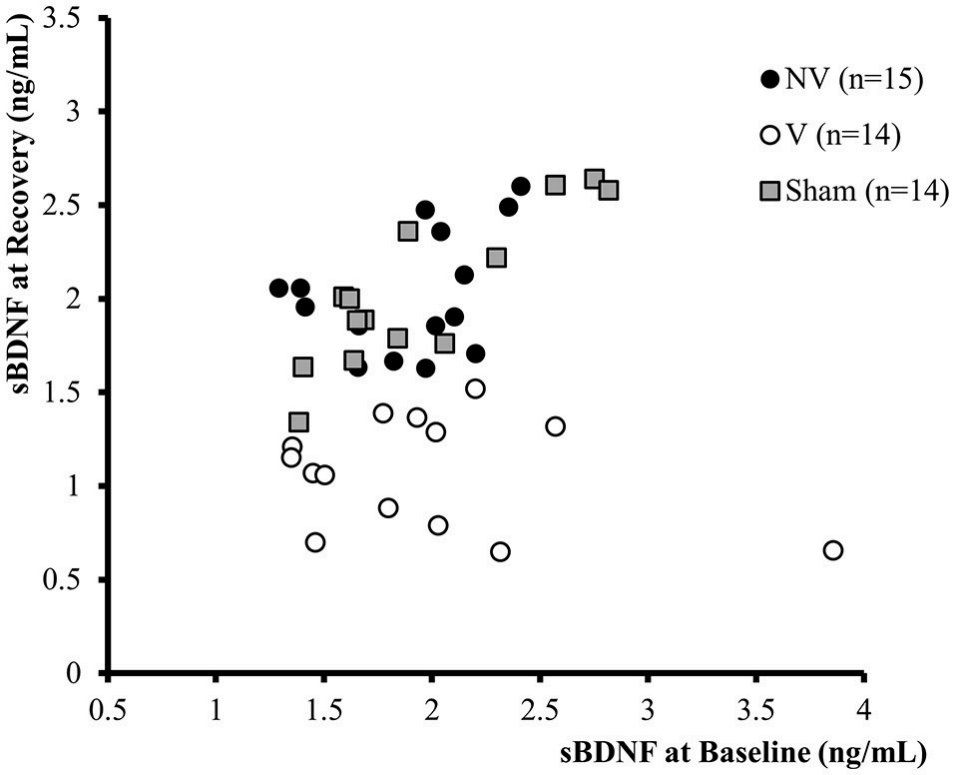
**Classification of animals according to the K-mean method based on Baseline (before SD—X axis) and Recovery (31 days after SD—Y axis)**. The sBDNF values are expressed in ng/mL for V (white dots, *n* = 14) and NV (black dots, *n* = 15) animals.

One month after SD, we identified vulnerable (V, *n* = 14) and non-vulnerable animals (NV, *n* = 15) using a k-mean method based on their sBDNF levels during Baseline and Recovery (Figure 2).

### Time-dependent evolution of sBDNF levels

**Figure 3:**
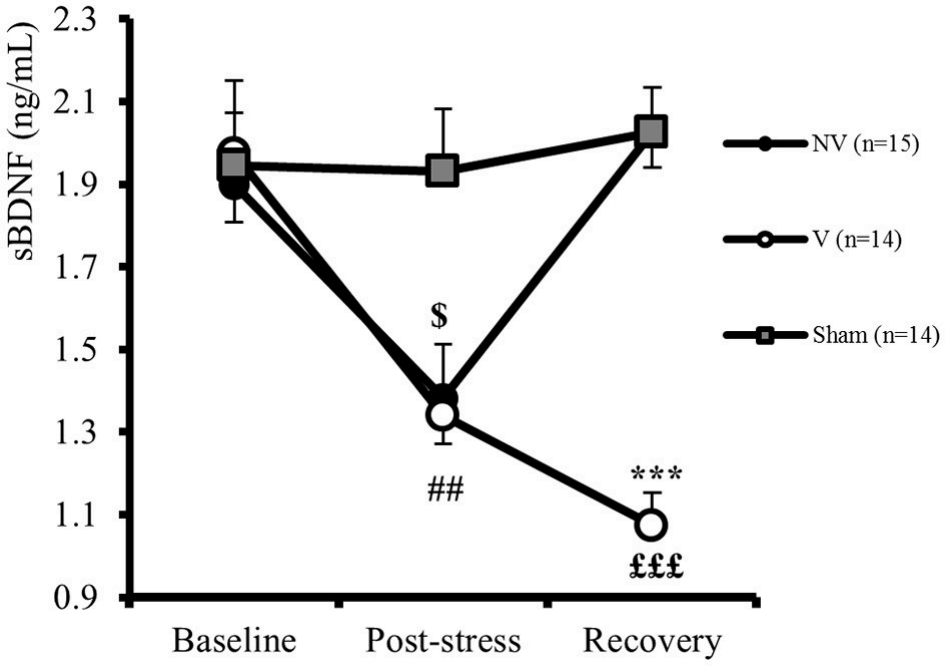
**Time course of sBDNF (ng/mL) in Sham (Sham, *n* = 14), Vulnerable (V, *n* = 14), and Non-Vulnerable (NV, *n* = 15) rats**. Statistical differences were evaluated by ANOVA for repeated measures between V and NV (Group^*^Time effect: *p* < 0.001) followed by *post-hoc* Bonferroni test. The results are expressed as following: between V and NV at Recovery: ^***^p < 0.001; for NV between Post-stress and Baseline: $*p* < 0.05; for V between Post-stress and Baseline: ##*p* < 0.01; for V between Recovery and Baseline: £££*p* < 0.001. Differences with Sham at each time were evaluated by factorial ANOVA at Baseline [*F*_(2, 40)_ = 0.08, ns], at Post-Stress [*F*_(2, 40)_ = 5.13, *p* < 0.05, *post-hoc* Bonferroni test: NV vs. Sham: *p* < 0.05, V vs. Sham: *p* < 0.05], and at Recovery [*F*_(2, 40)_ = 36.41, *p* < 0.001, *post-hoc* Bonferroni test: NV vs. Sham: ns and V vs. Sham: *p* < 0.001] (not shown on figure). Values are given as mean ± SEM.

The time course of sBDNF was different in NV and V rats (Figure 3; Supplementary Table 2, Group^*^Time effect: *p* < 0.001): sBDNF decreased after SD to return to Baseline values 1 month later in the NV group, but remained low in the V group.

### EEG features in vulnerable and non-vulnerable animals

**Figure 4:**
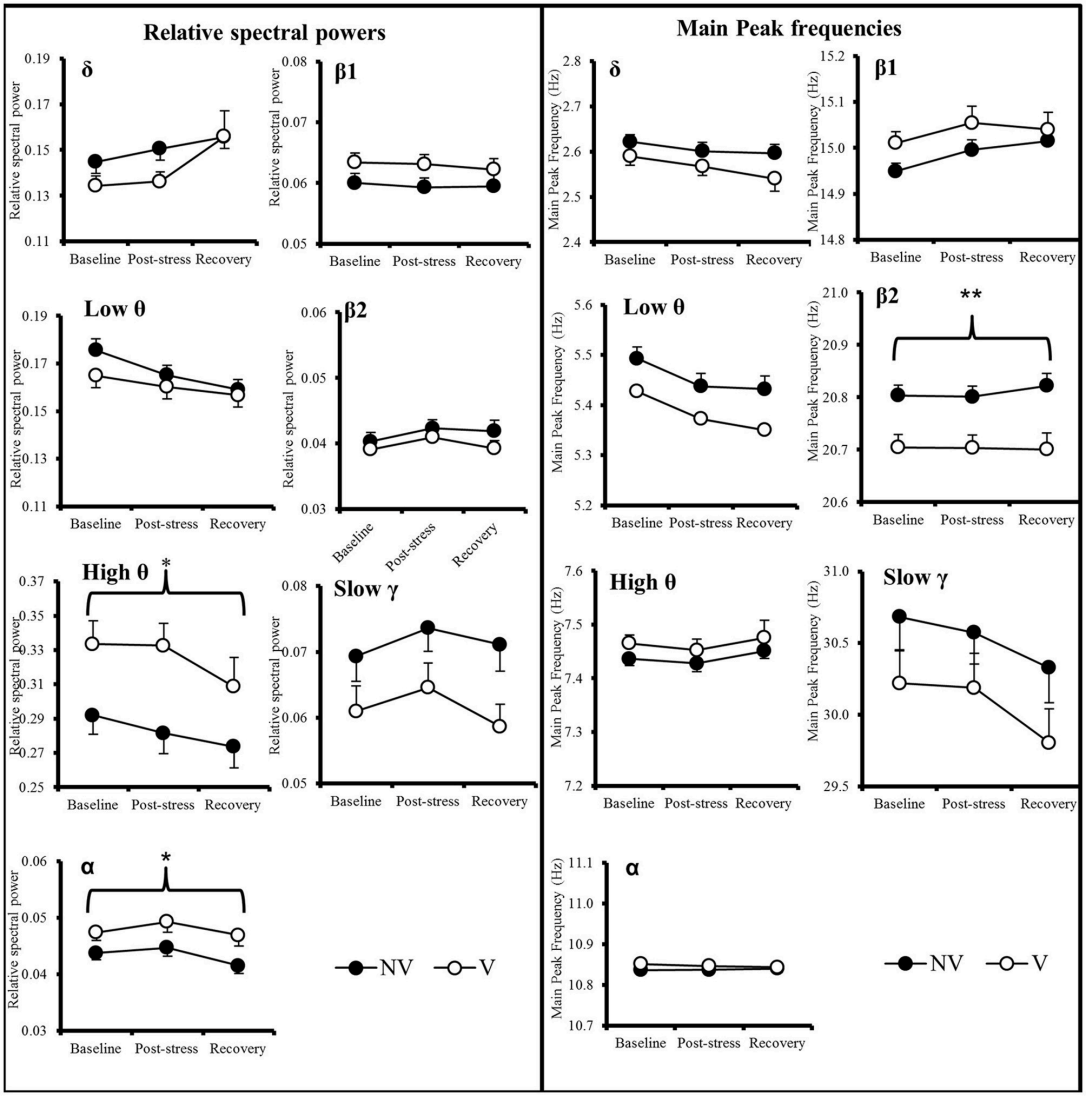
**Time course of the relative spectral power and main peak frequencies in δ, low θ, high θ, α, β1, β2, and slow γ in Vulnerable (*n* = 14) and Non-Vulnerable (*n* = 15) rats**. Comparisons were carried out using ANOVA for repeated measures followed if necessary by *post-hoc* Bonferroni tests. The significant Group effects between NV and V are expressed as: ^*^p < 0.05; ^**^p < 0.001 above the horizontal brace spanning the three time points. Values are given as mean ± SEM.

In order to determine whether EEG patterns can be used as predictive biomarkers, we compared V and NV animals at different time points before and after SD. Data from the sham group (which contains an unidentifiable number of potential V and NV animals) are presented in Supplemental information (Supplementary Figure 4). All significant results of repeated measure ANOVA are reported in Supplementary Table 3. The relative spectral power and the main peak EEG frequency (Figure 4, Supplementary Table 3) of each band were stable across time within each NV and V group. However, as compared to NV rats, V animals were characterized by greater relative power levels in the high θ and α bands and by a lower main peak frequency in the β2 band (Group effect: *p* < 0.05 for high θ and α bands and *p* < 0.001 for β2 main peak frequency, Supplementary Table 3).

**Figure 5:**
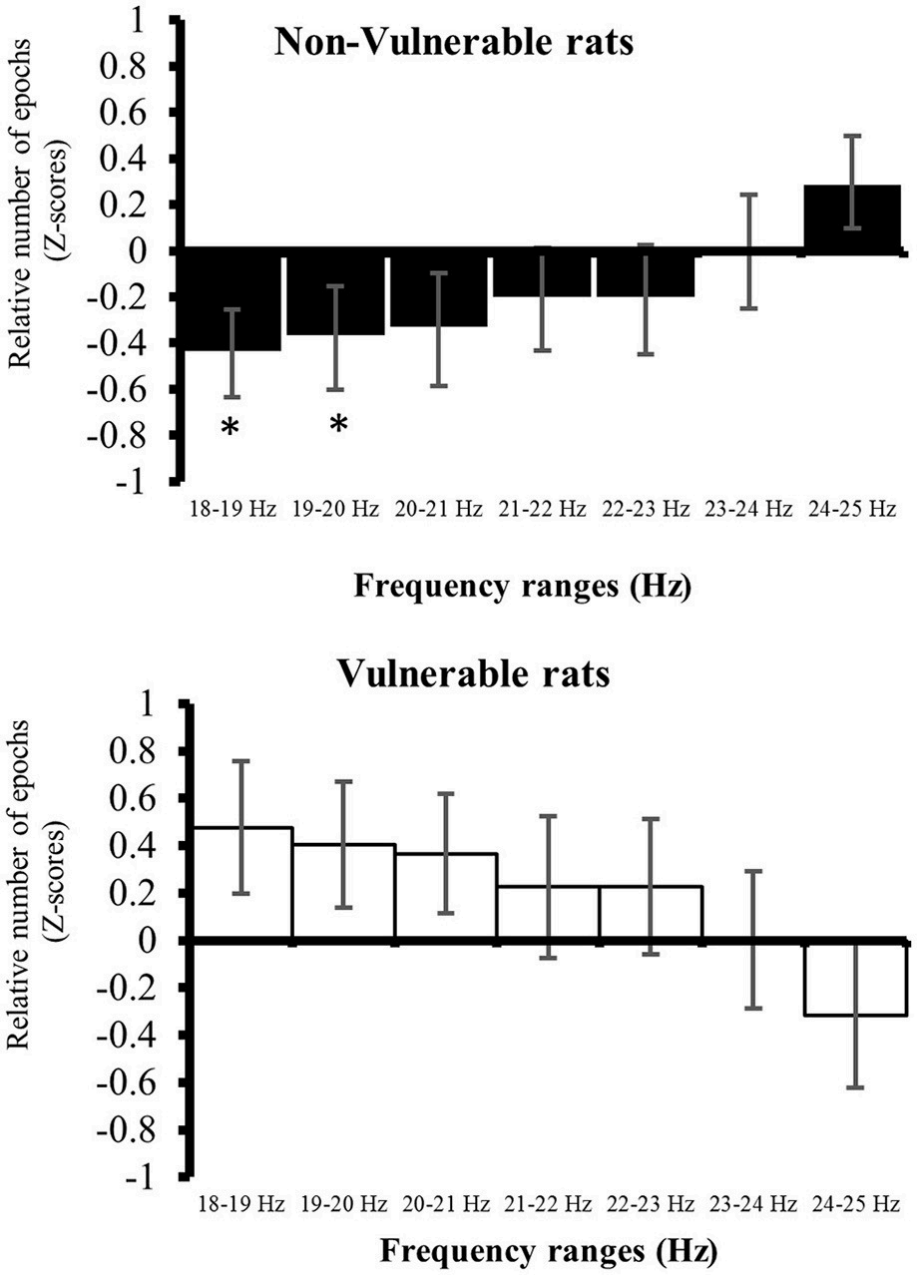
**Repartition of the relative number of epochs in the β2 band during 22 h of active wake at Baseline**. Comparisons at a given interval frequencies were carried out using factorial ANOVA. The results are expressed as: ^*^p < 0.05. Values are given as mean ± SEM.

Differences between V and NV at Baseline for the main peak frequency in the β2 band were characterized by a higher repartition into lower values of the β2 frequency for V compared to NV animals [Figure 5; 18–19Hz: *F*_(1, 27)_ = 7.62 *p* < 0.05; 19–20Hz: *F*_(1, 27)_ = 5.12, *p* < 0.05].

### Biomarkers of vulnerability to depression

We investigated whether high θ and α relative powers and ß2 main peak frequency can be used as predictive biomarker of vulnerability to depression. To this aim, we calculated the Area Under the Curve (AUC) of the Receiver Operating Characteristic (ROC).

**Figure 6:**
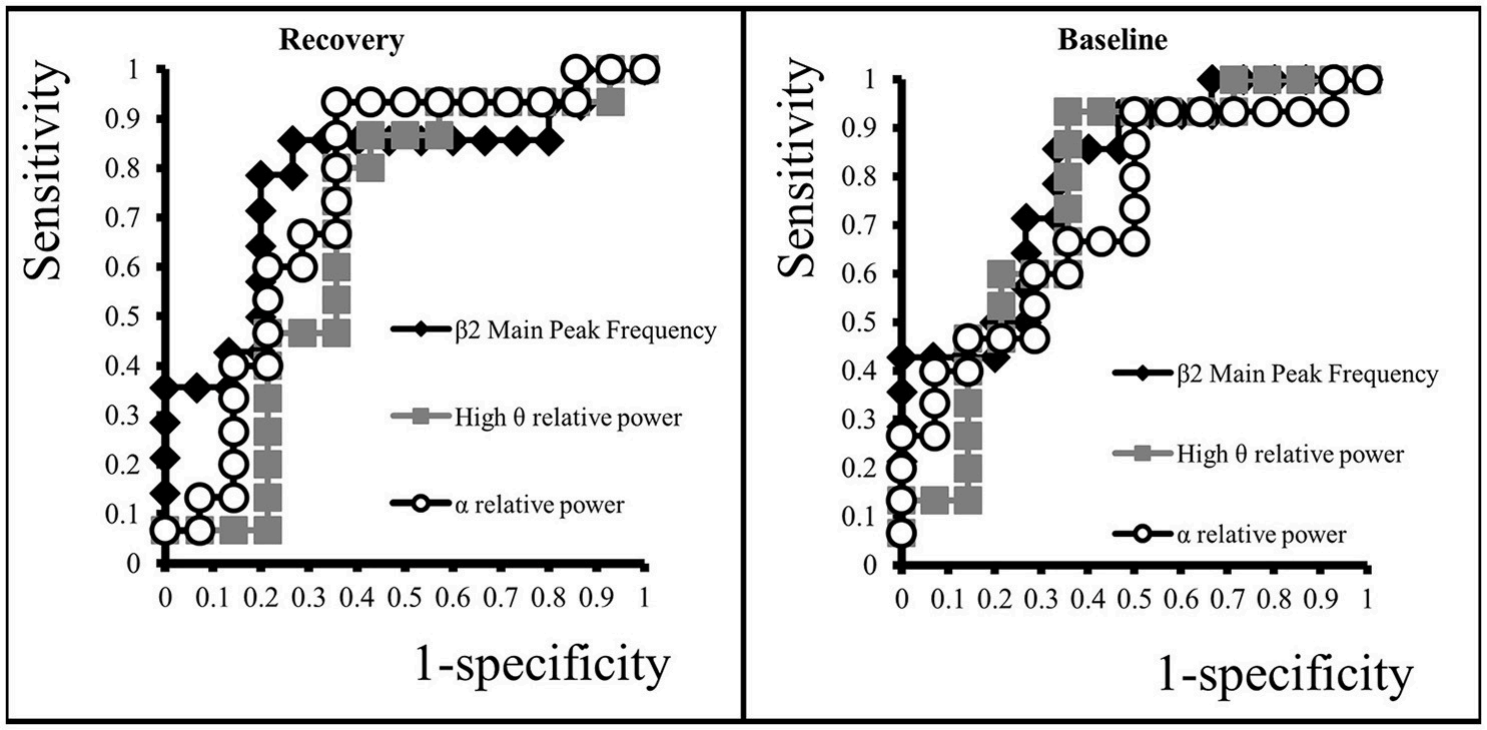
**ROC Curves of β2 main peak frequency, high θ and α relative spectral power at Recovery and Baseline**.

At Recovery, α relative power was a good predictor of vulnerability with an AUC of 0.74 (CI_95_: 0.55–094, *p* < 0.05), but high θ power was not predictive with an AUC of 0.67 (CI_95_: 0.45–0.88, *p* = 0.11). However, we found that a low ß2 main peak frequency was a better predictor with an AUC of 0.78 (CI_95_: 0.60–0.96, *p* < 0.01) (Figure 6). At Baseline, low ß2 main peak frequency also yielded a very good ability to predict V rats with an AUC of 0.80 (CI_95_: 0.64–0.96, *p* < 0.01; Figure 6). High relative powers in the high θ and α bands were also predictive with an AUC of 0.76 (CI_95_: 0.57–0.94, *p* < 0.05) and 0.72 (CI_95_: 0.54–0.91, *p* < 0.05), respectively.

The relationship between the β2 main peak frequency and vulnerability to depression is better apparent when correlating Recovery sBDNF and β2 main peak frequency at Baseline or Recovery. A slight correlation exists between Recovery sBDNF levels and Baseline β2 main peak frequency (*r* = 0.43, *r*^2^ = 0.18, *p* < 0.05) as well as for Recovery β2 main peak frequency (*r* = 0.41, *r*^2^ = 0.17, *p* < 0.05) (Supplementary Figure 5). However, there was no correlation between high θ or α relative powers and sBDNF levels at Baseline or Recovery.

## Discussion

In the present study, we report that the main peak frequency in the β2 band can be used as a predictive biomarker of vulnerability to depression, before the occurrence of a major stressful event.

### Heterogeneity in animals

As previously reported (Blugeot et al., 2011; Becker et al., 2015; Bouvier et al., 2016) we confirm that an intense stress splits the animal population into two groups: those recovering to pre-stress sBDNF levels (non-vulnerable to depression) and those that did not (vulnerable to depression). This demonstrates that (i) the animals are not equivalent before social defeat, as they will react differently; and (ii) an intense stress is a prerequisite to identify a vulnerable population using sBDNF level. This also indicates that laboratory animals have different diathesis, which could stem from different genetic/epigenetic background, different handling conditions by the dam, by the breeder, different access to food, different social interactions, etc. (Bernard, 2016). Taking advantage of this heterogeneity, we searched for biomarkers identifying the future NV and V populations, before the occurrence of a major stressful event.

### EEG features as a marker of vulnerability to depression

We report that future V and NV rats show differences in the β2 main peak frequency and in high θ and α relative powers. Before any manipulation, animals can thus be differentiated on the basis of their EEG properties. Since differences in main peak EEG frequencies between V and NV animals were not associated with difference in main peak detection (Supplementary Table 1), these effects were not due to the detection algorithm. Changes in locomotion activity were also unlikely contributing to EEG modifications during active wake (Supplementary Figure 2), since we did not observe any difference in the time course of locomotion. Finally, we did not observe any difference on circadian variations of core body temperature (Supplementary Figure 1), suggesting that EEG differences are unlikely due to circadian alterations.

Although, θ power was a good predictor of vulnerability at Baseline, it was not at Recovery. It is possible that the properties of θ oscillations at Recovery were modified due to the various morphofunctional alterations that occur in V animals, which may disrupt rhythm generators (Wulff et al., 2009; Ducharme et al., 2012). Alternatively, an insufficient statistical power due to the limited group size cannot be rule out.

We also observed a change in power and frequency during the course of the experiment. When long-term electrophysiological recordings are performed, the quality of the signal may degrade over time (growth of tissue, glial scar, etc.). This consistently translates in a large decrease in the contribution of fast oscillations, which are the first to be filtered out (Worrell, 2012). In our experimental conditions, the power in the slow γ band remained stable between Baseline and Recovery in all groups, demonstrating that changes in power cannot be ascribed to signal loss. Supradural EEG recordings, as performed here, represent a crude image of EEG activity, mostly volume conducted (Delorme et al., 2012), and highly filtered (Worrell, 2012). Hence, identifying modifications as shown here is quite a remarkable achievement, as it opens the way to non-invasive recordings in humans with scalp EEG recordings. Future studies are required to assess the origin of these oscillatory activities.

### Comparison with human studies

In humans, depression and vulnerability to depression are not associated to clear EEG biomarkers, except for the hemispheric asymmetry in the α band for depressive (Reid et al., 1998; Kentgen et al., 2000) and vulnerable (Bruder et al., 2005) subjects, and the combination of two EEG factors, one related to brain deactivation and the other to brain activation (Grin-Yatsenko et al., 2010). However, the main peak frequencies in the different frequencies bands remain to be analyzed in humans. The frequency of oscillations is a parameter worth considering since it is barely affected by the changes in recording conditions and by the filtering effects of the slap, bone etc.

### Possible mechanisms

At present, it is not possible to determine whether there exists a causal relationship between different EEG properties before SD and sBDNF levels in V and NV animals at Recovery. However, we observed a correlation between sBDNF Recovery levels and β2 main peak frequency at Baseline or Recovery. It is interesting to note that BDNF polymorphism is accompanied by difference in EEG (Bulgin et al., 2008) and polysomnography in humans (Guindalini et al., 2014). The fact that sBDNF levels evolve differently in V and NV animals may be related to BDNF polymorphism.

The mechanisms accounting for different β2 main peak frequencies in future V and NV animals remain to be investigated. It is interesting to note that bicuculline, a GABA_A_ antagonist, shifts the β2 peak frequency toward low frequency (Arai and Natsume, 2006), and increases θ and α powers (Butuzova and Kitchigina, 2008) as we observed in our V animals. Different functional architectures in GABAergic circuits may explain the differences in β2 peak frequency in future V and NV animals.

### EEG as an early predictor of depression vulnerability

Our results indicate that EEG features can be used to predict future V and NV animals, with a better predictability for low β2 main peak frequency. One apparent limitation of the present study is that an intense stressor is necessary to identify V and NV animals based on sBDNF levels. This protocol was used to extract the appropriate EEG biomarkers. However, identifying an at-risk population with a ROC AUC of 0.8 represents a remarkable achievement that opens the way to human studies.

The EEG marker also identifies the vulnerability to depression *after* SD during the Recovery period. But the main originality of the present work is to demonstrate that the same EEG marker can be used to identify an at-risk population before the major stressful event that will bring them close to the threshold to depression. Studying differences in β2 main peak frequency in other stress-related mental disorders such as Post-Traumatic Stress Disorder, anxiety, etc. would be important to understand the general value of this biomarker.

## Conclusion

We here provide evidence that EEG analysis of active wake can be used to identify future vulnerability to depression in rats. Thus, EEG could be a helpful tool to explore mechanisms of depression vulnerability. Given the straightforwardness of scalp EEG recordings in humans, our results bear a potential translational value.

## Author contributions

Conception and design of the study: DC, CBec, CBer, JB, and FCan. Acquisition and analysis of data: DC, CBec, AG, MC, FCam, CBec, JB, and FCan. Drafting a significant portion of the manuscript or figures: DC, CBec, CBer, JB, and FCan. Revising the work critically for important intellectual content: DC, CBec, AG, MC, FCam, CBer, JB, and FCan. Final approval of the version to be published: DC, CBec, AG, MC, FCam, CBer, JB, and FCan. Agreement to be accountable for all aspects of the work in ensuring that question related to the accuracy or integrity of any part of the work are appropriately investigated and resolved: DC, CBec, AG, MC, FCam, CBer, JB, and FCan.

## Funding

Grants from the Délégation Générale à l'Armement (DGA), Fédération pour la Recherche sur le Cerveau (FRC), the Institut National de la Santé et de la Recherche Médicale (INSERM), and the Université Pierre et Marie Curie (UPMC) supported the study.

### Conflict of interest statement

The authors declare that the research was conducted in the absence of any commercial or financial relationships that could be construed as a potential conflict of interest. The opinions or assertions expressed here in are the private views of the authors and are not to be considered as official or as reflecting the views of the French Military Health Service.
