# Supplementary material for: Low β2 Main Peak Frequency in the Electroencephalogram Signs Vulnerability to Depression
Source: Front Neurosci. 2016 Nov 2;10:495. doi: 10.3389/fnins.2016.00495 (PMC5090000; doi:10.3389/fnins.2016.00495)
Supplement: Supplementary file 1 [file Table1.PDF]

**Supplementary Table 1: Values and factorial ANOVA results for epochs of active wake with a power peak detected.** The method for detecting power peaks returned the same number of peaks among all the 5-s epochs for V and NV animals at Baseline, Post-stress and Recovery in each band except for δ and High θ bands where more peaks were detected for V animals.

| | **Baseline** | | | **Post-stress** | | | **Recovery** | | |
|---|---|---|---|---|---|---|---|---|---|
| | **Sham** | **V** | **NV** | **Sham** | **V** | **NV** | **Sham** | **V** | **NV** |
| **δ (1.5-4 Hz)** | 1718±84 | 1742±62 | 1550±43 | 1515 ±70 | 1793±79 | 1659±62 | 1406±61 | 1464±75 | 1305±69 |
| | | **F(1,27)=6.64, p<0.05** | | | F(1,27)=1.81, ns | | | F(1,27)=2.39, ns | |
| **Low θ (4-6.5 Hz)** | 1067±70 | 1034±83 | 1075±50 | 915±52 | 1021±88 | 1166±58 | 849±41 | 896±69 | 948±62 |
| | | F(1,27)=0.18, ns | | | F(1,27)=1.95, ns | | | F(1,27)=0.32, ns | |
| **High θ (6.5-9.5 Hz)** | 2008±102 | 2073±51 | 1845±41 | 1790±82 | 2151±76 | 2075±81 | 1747±83 | 1807±87 | 1677±76 |
| | | **F(1,27)=12.44, p<0.01** | | | F(1,27)=0.47, ns | | | F(1,27)=1.28, ns | |
| **α (9.5-12 Hz)** | 1334±68 | 1329±60 | 1294±31 | 1172±55 | 1359±79 | 1424±54 | 1075±44 | 1174±61 | 1140±53 |
| | | F(1,27)=0.28, ns | | | F(1,27)=0.47, ns | | | F(1,27)=0.18, ns | |
| **β1 (13-18 Hz)** | 2431±111 | 2441±70 | 2264±55 | 2113±80 | 2490±109 | 2486±85 | 2005±79 | 2083±99 | 2009±95 |
| | | F(1,27)=4.00, ns | | | F(1,27)=0.001, ns | | | F(1,27)=0.29, ns | |
| **β2 (18-25 Hz)** | 2463±113 | 2470±72 | 2293±57 | 2139±80 | 2518±111 | 2514±86 | 2027±80 | 2104±100 | 2032±96 |
| | | F(1,27)=3.77, ns | | | F(1,27)=0.001, ns | | | F(1,27)=0.27, ns | |
| **Slow γ (25-48 Hz)** | 2467±113 | 2471±72 | 2294±57 | 2141±81 | 2520±111 | 2515±86 | 2029±80 | 2107±100 | 2034±96 |
| | | F(1,27)=3.77, ns | | | F(1,27)=0.001, ns | | | F(1,27)=0.28, ns | |
