# Supplementary material for: Low β2 Main Peak Frequency in the Electroencephalogram Signs Vulnerability to Depression
Source: Front Neurosci. 2016 Nov 2;10:495. doi: 10.3389/fnins.2016.00495 (PMC5090000; doi:10.3389/fnins.2016.00495)
Supplement: Supplementary file 2 [file Table2.PDF]

Supplementary Table 2: Repeated ANOVA results for sBDNF values.

	Repeated ANOVA results for sBDNF values
	V *vs.* NV
**ANOVA effects**	**Group*Time effect: F(2,54)=14.31 (p<0.001)**<br>**Group effect: F(1,27)=5.96 (p<0.05)**<br>**Time effect: F(2,54)=15.47 (p<0.001)**
Post hoc Bonferroni test results	
**NV**	Baseline *vs.* Post-stress: p<0.05<br>Post-stress *vs.* Recovery: p<0.001
**V**	Baseline *vs.* Post-stress: p<0.01<br>Baseline *vs.* Recovery: p<0.001
**V *vs.* NV**	NV Baseline *vs.* V Post-stress: p<0.05<br>NV Baseline *vs.* V Recovery: p<0.001<br>NV Post-stress *vs.* V Baseline: p<0.05<br>NV Recovery *vs.* V Post-stress: p<0.01<br>NV Recovery *vs.* V Recovery: p<0.001
