# Supplementary material for: Low β2 Main Peak Frequency in the Electroencephalogram Signs Vulnerability to Depression
Source: Front Neurosci. 2016 Nov 2;10:495. doi: 10.3389/fnins.2016.00495 (PMC5090000; doi:10.3389/fnins.2016.00495)
Supplement: Supplementary file 3 [file Table3.PDF]

Supplementary Table 3: Repeated ANOVA results for relative spectral power or main peak frequencies.

	Repeated ANOVA results for Relative Spectral Power<br>V *vs.* NV	Repeated ANOVA for Main Peak Frequencies<br>V *vs.* NV
**δ (1.5-4Hz)**	Time effect : F(2,54)=7.00 (p<0.01)	Time effect : F(2,54)=4.50 (p<0.05)
	Post hoc Bonferroni test :<br>Baseline vs Recovery : p<0.01<br>Post-stress vs Recovery : p<0.05	*Post hoc Bonferroni test :*<br>Baseline vs Recovery : p<0.05
**Low θ (4-6.5Hz)**	Time effect : F(2,54)=13.99 (p<0.001)	Time effect : F(2,54)=9.27 (p<0.001)
	Post hoc Bonferroni test :<br>Baseline vs Post-stress : p<0.01<br>Baseline vs Recovery : p<0.001	*Post hoc Bonferroni test :*<br>Baseline vs Post-stress : p<0.01<br>Baseline vs Recovery : p<0.001
**High θ (6.5-9.5Hz)**	**Group effect : F(1,27)=6.14** (p<0.05)<br>Time effect : F(2,54)=6.10 (p<0.01)	ns
	Post hoc Bonferroni test :<br>Baseline vs Recovery : p<0.01	
**α (9.5-12Hz)**	**Group effect : F(1,27)=5.24** (p<0.05)<br>Time effect : F(2,54)=6.43 (p<0.01)	ns
	Post hoc Bonferroni test :<br>Post-stress vs Recovery : p<0.01	
**β1 (13-18Hz)**	ns	Time effect : F(2,54)=6.54 (p<0.01)
		Post hoc Bonferroni test :<br>Baseline vs Post-stress : p<0.05<br>Baseline vs Recovery : p<0.01
**β2 (18-25Hz)**	Time effect : F(2,54)=3.98 (p<0.05)	**Group effect : F(1,27)=15.24 (p<0.001)**
	Post hoc Bonferroni test :<br>Baseline vs Post-stress : p<0.05	
**Slow γ (25-48Hz)**	Time effect : F(2,54)=5.03 (p<0.01)	Time effect : F(2,54)=10.29 (p<0.001)
	Post hoc Bonferroni test :<br>Baseline vs Post-stress : p<0.05<br>Post-stress vs Recovery : p<0.05	*Post hoc Bonferroni test :*<br>Baseline vs Recovery : p<0.001<br>Post-stress vs Recovery : p<0.01
