# Supplementary figures and images for: Low β2 Main Peak Frequency in the Electroencephalogram Signs Vulnerability to Depression
Source: Front Neurosci. 2016 Nov 2;10:495. doi: 10.3389/fnins.2016.00495 (PMC5090000; doi:10.3389/fnins.2016.00495)

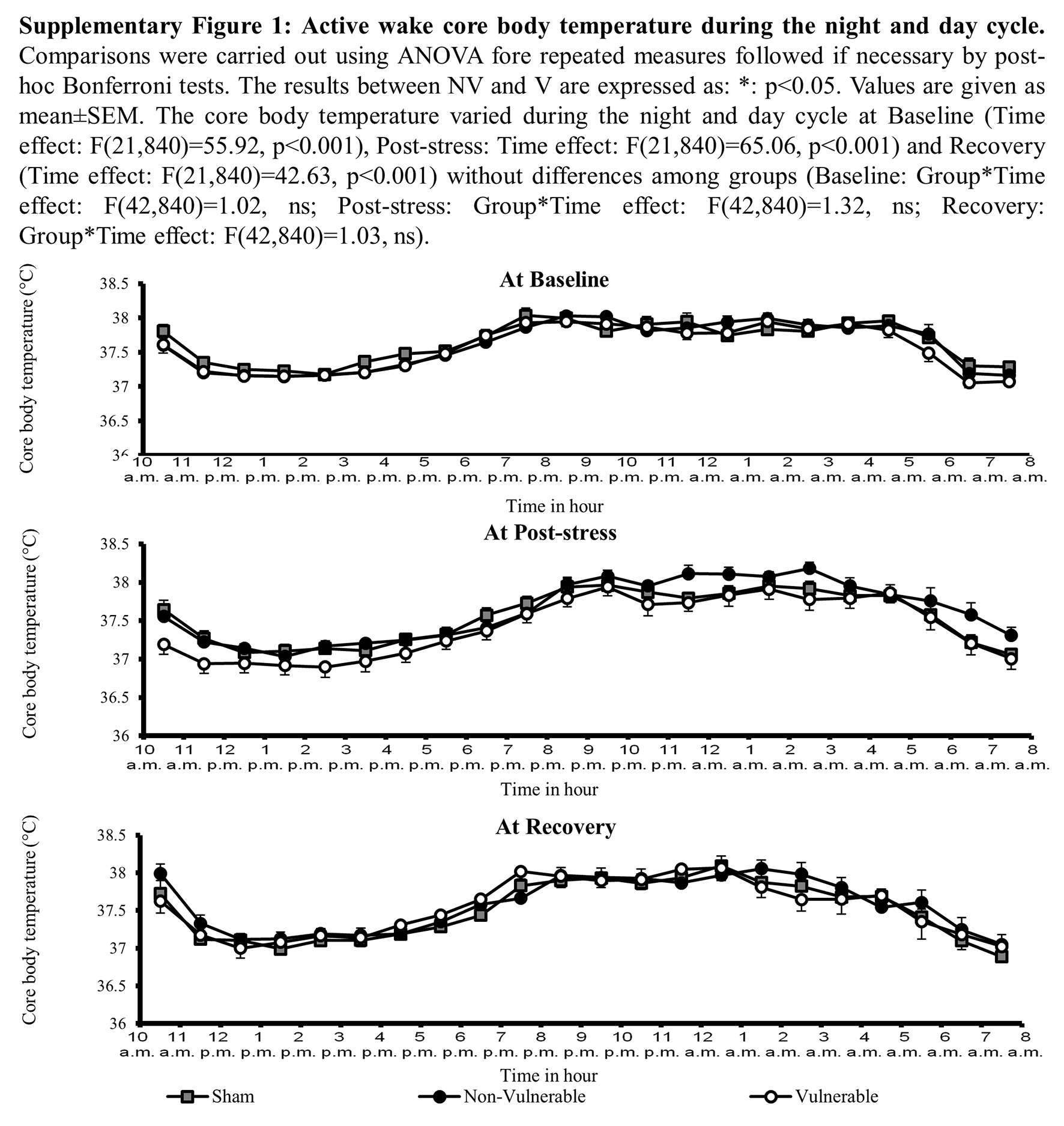

Supplement: Supplementary file 4 [file Image1.TIF]

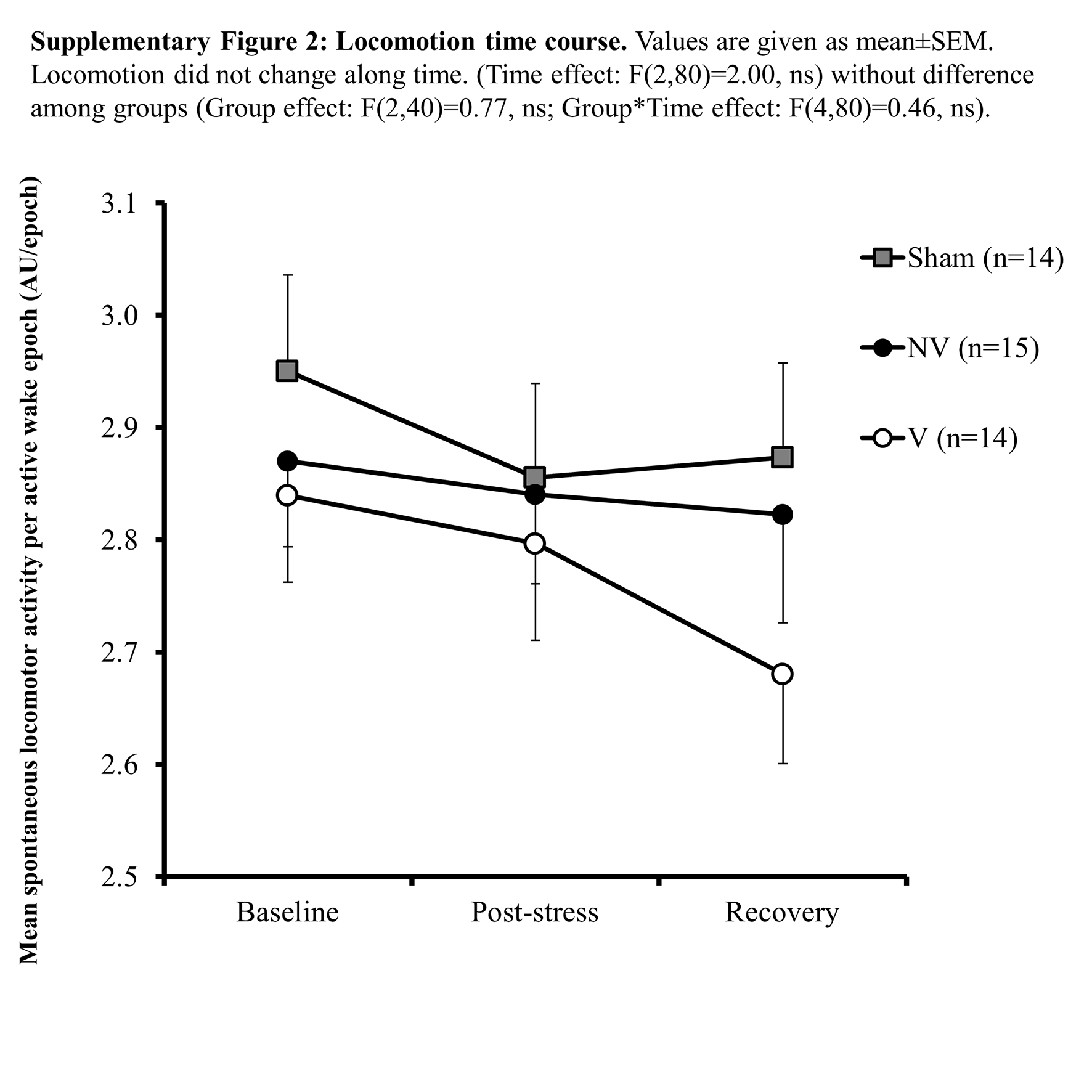

Supplement: Supplementary file 5 [file Image2.TIF]

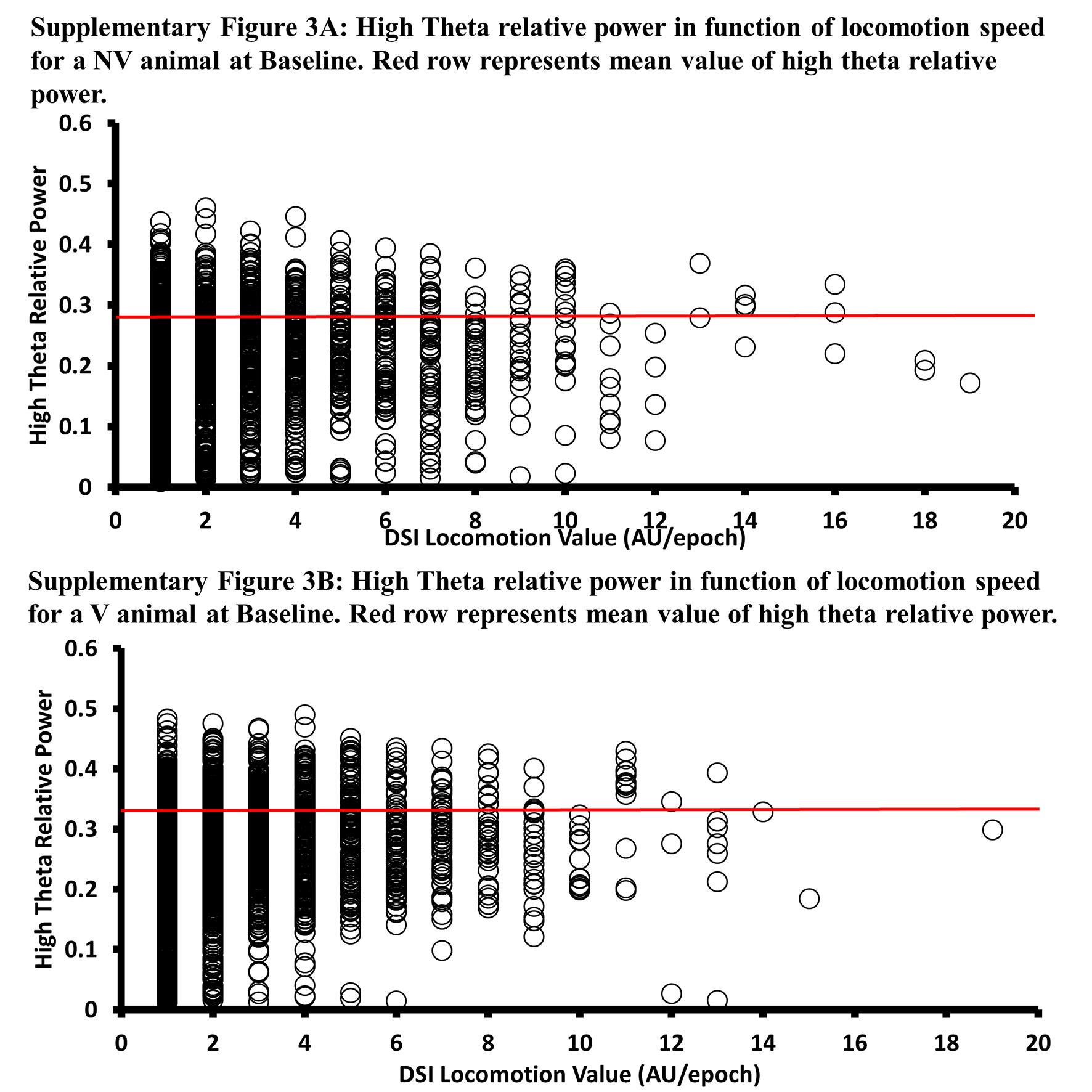

Supplement: Supplementary file 6 [file Image3.TIF]

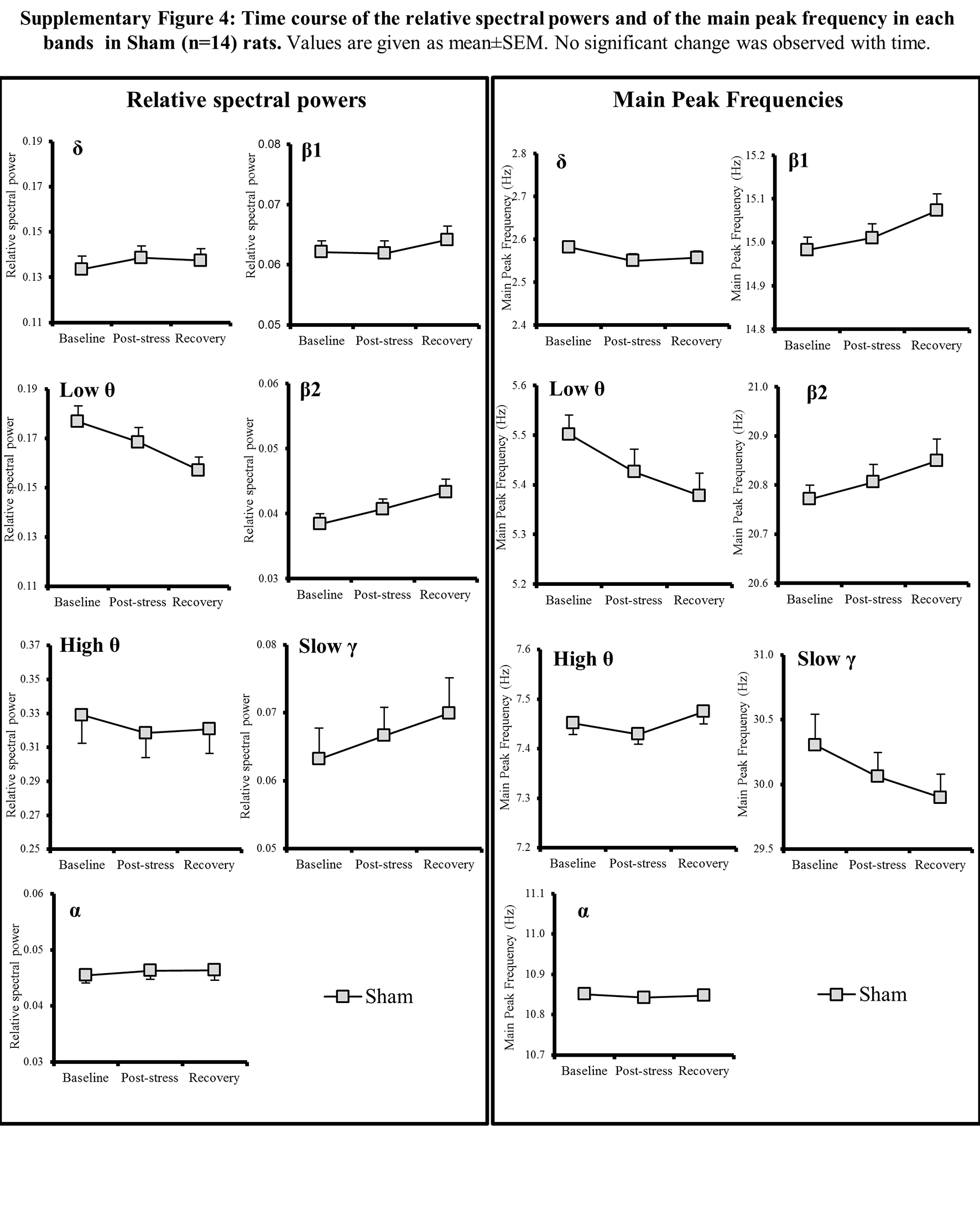

Supplement: Supplementary file 7 [file Image4.TIF]

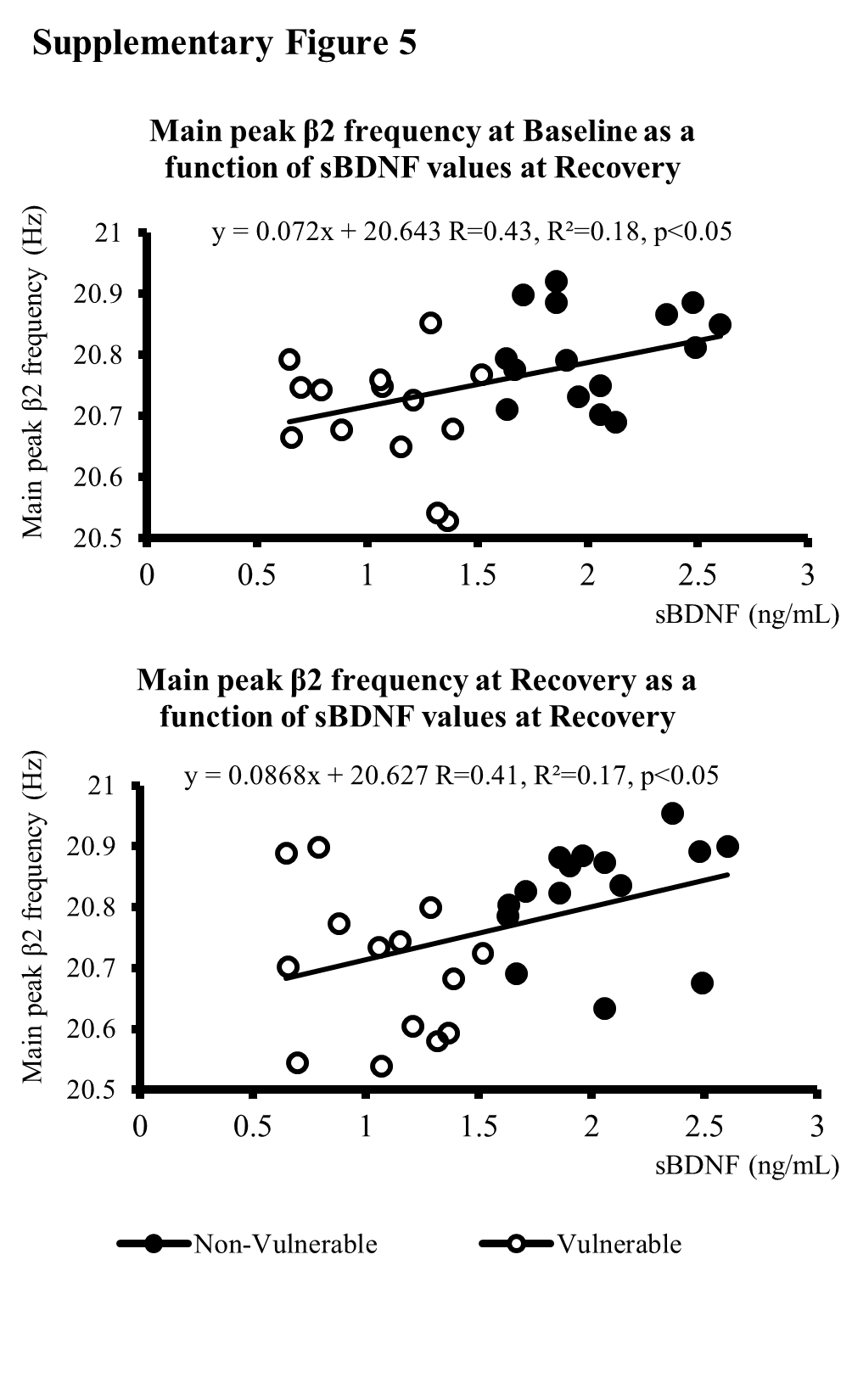

Supplement: Supplementary file 8 [file Image5.TIF]
